# Less hip range of motion is associated with a greater alpha angle in people with longstanding hip and groin pain

**DOI:** 10.1007/s00167-021-06733-2

**Published:** 2021-09-12

**Authors:** August Estberger, Anders Pålsson, Ioannis Kostogiannis, Eva Ageberg

**Affiliations:** 1 Department of Health Sciences, Lund University, Lund, Sweden; 2 Department of Orthopaedics, Clinical Sciences, Lund University, Lund, Sweden

**Keywords:** Alpha angle, Femoroacetabular impingement, Cam morphology, Range of motion

## Abstract

**Purpose:**

A higher alpha angle has been proposed to correlate with lower hip range of motion, but the association in people with longstanding hip and groin pain is currently unclear. The aims were to: (1) assess the association between range of motion and alpha angle in patients with longstanding hip and groin pain; (2) examine if a cut-off value in range of motion variables could identify patients with an alpha angle above or below 60°.

**Methods:**

Seventy-two participants were consecutively recruited from an orthopaedic department after referral for hip- and groin-related pain. Passive hip range of motion was measured in flexion, internal rotation with 90° hip flexion, internal rotation in neutral hip position, external rotation with 90° hip flexion, and abduction. The alpha angle was calculated from a frog-leg lateral radiograph. Linear regression examined the association between range of motion and alpha angle, and an ROC-curve analysis was performed to identify the sensitivity and specificity of range of motion cut-offs.

**Results:**

Lower range of motion in internal rotation in flexion, external rotation, and abduction were associated with higher alpha angle. Internal rotation of 27° or less displayed good sensitivity (81%) and specificity (85%) to detect an alpha angle above 60°, while a cut-off of 41° in external rotation and 27° in abduction showed a sensitivity of 72% and specificity of 50% and 60%, respectively.

**Conclusion:**

Less internal rotation in flexion, external rotation, and abduction are associated with a greater alpha angle in a cohort of people with longstanding hip and groin pain. A cut-off of 27° in internal rotation has good sensitivity and specificity to identify people with an alpha angle above or below 60° and have the potential to be used in the clinical setting to identify patients that require further imaging, or that are unlikely to have cam morphology.

**Level of evidence:**

II.

## Introduction

Longstanding hip and groin pain is a common problem in young to middle-aged adults, leading to reduced participation in sports and activities of daily living, and reduced quality of life [16, 33]. Societal costs, in lost productivity and medical treatment, are also substantial [12, 30].

The hip joint is recognised as a source of groin pain, and femoroacetabular impingement syndrome (FAIS) is a common source of hip-related pain in a younger and physically active population [17]. Cam-type FAIS is currently understood to be the more severe of the morphological variations, with more associated labrum and cartilage damage [8, 15], as well as an increased risk of development of secondary hip osteoarthritis (OA) with greater cam deformities [21]. Cam morphology is commonly quantified by the alpha angle, defined as the angle between lines from the center of the head of the femur to the longitudinal axis and the neck of the femur and the deviation of the spherical nature of the femoral head [32]. Although cam morphology is common in the general population [14], greater deformities are seen in athletes and symptomatic populations [14, 29]. Various cut-offs of the alpha angle have been used in the literature to define cam morphology, but a recent systematic review concluded that 60° is currently the most appropriate cut-off [23].

Cam morphology has been proposed to affect hip ROM, primarily in positions of hip flexion and internal rotation [11]. Several studies have examined the effect of the alpha angle on hip ROM in asymptomatic people and found less ROM in participants with higher alpha angles as compared to those with lower alpha angles, primarily in internal rotation with 90° hip flexion [18, 19, 22, 27, 31]. However, the relationship between alpha angle and ROM in symptomatic people is conflicting, with one systematic review concluding that people with FAIS did [7], and another concluding that these people did not [10] have less ROM compared to asymptomatic controls. Despite the existing conflicting evidence, ROM restriction is commonly used as a diagnostic criterion for FAIS [13]. Examining the association of cam morphology and ROM in symptomatic people can provide information on the value of ROM assessment in differential diagnosis of hip and groin pain.

The diagnosis of FAIS is based on clinical findings, patient-reported symptoms and radiographic imaging [13]. However, not all clinicians, such as physical therapists and primary care practitioners, have immediate access to imaging. In addition, the use of ionising radiation is associated with risks [39] and societal costs. A clinical tool that can identify patients that are likely to have cam morphology could be of benefit for clinicians in deciding who to refer for radiographic investigation. However, there is currently only low-level evidence regarding the diagnostic accuracy of clinical tests to identify cam morphology [2].

The aim of this study was to examine the association between passive hip range of motion and alpha angle in patients with longstanding hip and groin pain. A secondary aim was to examine whether a cut-off value in ROM variables could identify patients with alpha angles above or below the clinical cut-off of 60°. The hypothesis was that lower range of motion would be associated with a higher alpha angle.

## Material and methods

This cross-sectional study was reported in accordance with the Strengthening of Observational Studies in Epidemiology (STROBE) statement (http://www.strobe-statement.org). Approval for the study was granted by the Regional Ethical Review Board in Lund (reg. no 2014/12) and the participants signed an informed consent form.

### Participants

**Fig. 1:**
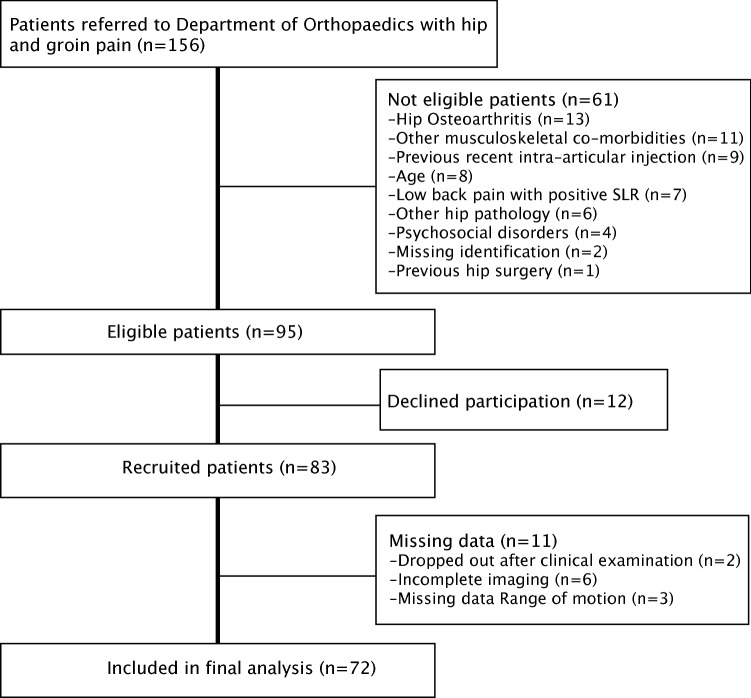
Recruitment flowchart

Participants were consecutively recruited from the Department of Orthopaedics at Skåne University Hospital, Sweden, between 2014 and 2017 as previously described [35] (Fig. 1). All patients referred to, or seeking health care, at the Department of Orthopaedics due to hip/groin pain (*n* = 156) were screened for eligibility by the orthopaedic surgeon in charge of hip arthroscopy. Inclusion criteria were: age 18–55 years; unilateral or bilateral hip and/or groin pain > 3 months. Patients with verified moderate or severe osteoarthritis or any other musculoskeletal comorbidities overriding the hip and groin pain, or limitations preventing testing of ROM, were excluded as described [35]. Ninety-five patients of 156 were eligible, 83 were enrolled in the study, and 2 participants dropped out [35]. Six participants had missing or sub-par frog-leg lateral projections, and the alpha angle could not be calculated for these subjects. Three participants had missing data from the ROM examination due to equipment malfunction. A total of 72 participants were thus included in the final analysis (Fig. 1). For descriptive purposes, the participants reported on perceived symptoms using the Copenhagen Hip and Groin Outcome Score (HAGOS), a valid and reliable PROM for use in patients with non-arthritic hip and groin pain [43]. HAGOS consists of six subscales (Symptoms, Pain, Activities of daily living, Sports and recreation, Physical activity and Quality of life), which are scored from 0 to 100 with 100 representing no symptoms, and has been validated for use in Sweden [42]. Patient characteristics and HAGOS score are presented in Table 1.

**Table 1:** Patients characteristics and HAGOS score

	All patients (*n* = 72)	Alpha angle < 60° (*n* = 40)	Alpha angle ≥ 60° (*n* = 32)
Age (years)	35.0 (9.1)	35.7 (8.5)	34.2 (9.9)
BMI	24.8 (4.0)	23.9 (4.1)^a^	25.9 (3.5)^a^
Sex, % males	36 (50)	11 (31)^b^	25 (69)^b^
HAGOS Symptoms	57 (15)	59 (18)	57 (16)
HAGOS Pain	58 (17)	58 (18)	57 (16)
HAGOS Activity	64 (21)	63 (22)	64 (20)
HAGOS Sport	49 (23)	50 (21)	47 (25)
HAGOS Participation	31 (29)	30 (27)	34 (30)
HAGOS QOL	29 (15)	29 (13)	29 (16)

Values are mean (SD), except sex, which is *n* (%)

*QOL* Quality of life

^a^BMI was higher in the high alpha angle group (*p* = 0.033)

^b^Sex was unevenly distributed with more males in the high alpha angle group (*p* =  < 0.001). No statistically significant differences in age and HAGOS subscales (*p* =  > 0.05)

### Imaging

Plain radiographs were conducted in a frog-leg lateral projection. A senior consultant specialised in skeletal radiology, who was not involved in the care of the patients analysed all radiographs. Alpha angle measurements were made on digital plain radiographs (Sectra IDS7 system) by drawing two lines from the centre of the femoral head; one along the longitudinal axis of the femoral neck and the other to the point of deviation of the femoral neck from a circular template. The angle between those lines represents the alpha angle, as described by Clohisy et al. [5]. A 60° alpha angle was used as a clinical cut-off for group allocation, with values ≥ 60° representing the high alpha angle group (*n* = 32) and values < 60° representing the low alpha angle group (*n* = 40).

### Passive hip range of motion

Passive hip ROM was measured by an experienced physical therapist blinded to imaging data, using a digital inclinometer and a digital goniometer (Commander Echo [JTECH Medical, Salt Lake City, Utah, USA]). A second physical therapist assisted in the assessment by fixating the participants.

Participants were asked to abstain from vigorous activity or exercise 48 h prior to testing. Participants wore tight fitting shorts and shirts for the examination. Prior to measurements, a short warm up was performed, consisting of cycling for 5 min at a self-selected pace at 75 W, 5 min of dynamic movements (calf raises and squats), and static and dynamic stretches for hamstring, calf and adductor muscles.

Movements tested were flexion (FLEX), internal rotation in neutral hip position (IRN), internal rotation with 90° hip flexion (IRF), external rotation with 90° hip flexion (ERF), and abduction (ABD).

FLEX was measured with the participant in supine position on a bench with the contralateral leg fixed and the digital inclinometer attached to the lateral side of the thigh, 10 cm proximal to the joint line of the knee. The examiner passively moved the patient’s leg into maximal flexion without rotation or adduction/abduction. IRN was measured in prone position with the participant’s pelvis fixed and the digital inclinometer attached 10 cm proximal to the lateral malleolus. IRF and ER were measured with the participant sitting on a bench, with the digital inclinometer placed 10 cm proximal to the lateral malleolus. The examiner passively moved the leg into maximal internal and external rotation. The participant was asked to maintain even pressure on the ischial tuberosities and a neutral pelvic tilt. ABD was measured in a supine position. The centre of a goniometer was placed over the ipsilateral ASIS. The stationary arm of the goniometer was aligned with the contralateral ASIS, and the moveable arm was aligned along the thigh to the centre of the patella. The examiner passively moved the leg into maximal abduction.

Each movement was measured twice, and the mean of these measurements served as the outcome measure [37]. All tests were performed on the right side first to randomise the order in which the affected side was tested. Each movement was taken to the end range of available joint motion, as perceived by the examiner. The patients were asked to report if the movement caused pain, and the examiner judged if end range of motion could be reached without muscle spasm or pain limiting range.

Excellent inter- and intra-rater reliability have previously been reported for hip ROM using digital inclinometry in healthy populations [24] and in people with hip OA [37]. A separate sample of healthy participants [*n* = 20, mean age (SD) 26 (7) years, 65% women] showed excellent intra-rater reliability (i.e. ICC > 0.8) for FLEX [ICC 0.839 (95% CI 0.643–0.933)], IRF [ICC 0.868 (95% CI 0.685–0.947)], IRN [ICC 0.941 (95% CI 0.859–0.976)], and ERF [ICC 0.842 (96% CI 0.641–0.934)]. ABD showed moderate intra-rater reliability (ICC > 0.6) [ICC 0.635 (95% CI 0.193–0.848)]. Passive extension was not included in the analyses due to poor intra-rater reliability (ICC < 0.2) [ICC 0.177 (95% CI − 0.293 to 0.571)]. The SEM was calculated for FLEX (4.3°), IRN (2.6°), IRF (3.6°), ERF (2.0°), and ABD (3.1°).

### Statistical analysis

SPSS version 25 (IBM Corp) was used for all statistical analysis. A linear regression model was used with alpha angle as the dependent variable and ROM as independent variables. An initial univariate linear regression was conducted for each ROM variable, and sex was later added to the model due to the uneven distribution of men in the high and low alpha angle groups. BMI also differed in the two groups, and age can influence hip ROM [25], but these variables did not reach statistical significance in our regression model and was therefore not adjusted for in further analysis. Multicollinearity was controlled for using variance inflation factors.

Student’s *t* tests and Fisher’s exact test were performed for numerical and categorical data, respectively, to determine between group differences in age, BMI, sex, and HAGOS scores.

Sensitivity and specificity were calculated by performing a ROC-curve analysis with the clinical cut-off at a 60° alpha angle and the ROM variables considered clinically and statistically relevant. The area under the curve (AUC) was determined to establish whether any ROM variables were suitable for predicting the alpha angle, with a value > 0.05 signifying predictive ability better than chance and a value of 1 indicating perfect predictive ability. The ROM threshold with the highest sensitivity and specificity to detect participants above or below the alpha angle cut-off was selected.

As this was a secondary analysis of the data sample, no a priori power calculation was conducted. As only two predictors (ROM variable and sex) was used in the models, the sample of *n* = 72 was considered sufficient, using the general rule of thumb of a minimum of 10 subjects per variable. Alpha was set at 0.05.

## Results

### ROM

There were significant differences in ROM in people with alpha angles above and below 60°, with the patients with higher alpha angles presenting with less ROM (Table 2). End range of motion could be reached for all participants.

**Table 2:** Range of motion, mean (SD) degrees and difference between groups

	All (*n* = 72)	< 60° (*n* = 40)	≥ 60° (*n* = 32)	*p* value
FLEX	99 (12)	102 (13)	95 (9)	0.010
IRF	28 (8)	33 (6)	21 (6)	0.000
IRN	39 (11)	44 (8)	32 (9)	0.000
ER	39 (8)	41 (7)	37 (8)	0.024
ABD	26 (6)	28 (5)	25 (6)	0.026

*IRF* internal rotation with 90° hip flexion, *IRN* internal rotation in neutral hip position, *FLEX* flexion, *ERF* external rotation with 90° hip flexion, *ABD* abduction

### Association between ROM and alpha angle

All ROM variables entered in the initial regression analysis were statistically significant. When adjusted for sex, FLEX and IRN displayed no significant predictive ability. IRF, ERF, and ABD predicted a higher alpha angle, with a 10° lower ROM predicting a higher alpha angle by 5.6° (95% CI 2.1°–9.2°) in IRF, 4.5° (95% CI 0.9°–8.0°) in ERF, and 5.4° (95% CI 0.6°–10.1°) in ABD, when adjusted for sex (Table 3).

**Table 3:** Regression models (unadjusted and adjusted)

Model unadjusted	*R*^2^	*B* (CI)	*p* value	Model adjusted for sex	*R*^2^	*B* (CI)	*p* value
FLEX	0.056	0.31 (0.04–0.58)	0.026	FLEX	0.276	0.15 (− 0.10 to 0.39)	n.s
IRF	0.262	0.86 (0.52–1.19)	< 0.001	IRF	0.353	0.56 (0.21 to 0.92)	0.003
IRN	0.198	0.61 (0.32–0.89)	< 0.001	IRN	0.293	0.29 (− 0.04 to 0.62)	n.s
ERF	0.127	0.66 (0.27–1.04)	0.001	ERF	0.323	0.45 (0.09 to 0.80)	0.014
ABD	0.084	0.74 (0.20–1.28)	0.008	ABD	0.312	0.54 (0.06 to 1.01)	0.027

*IRF* internal rotation with 90° hip flexion, *IRN* internal rotation in neutral hip position, *FLEX* flexion, *ERF* external rotation with 90° hip flexion, *ABD* abduction

### Sensitivity and specificity

**Fig. 2:**
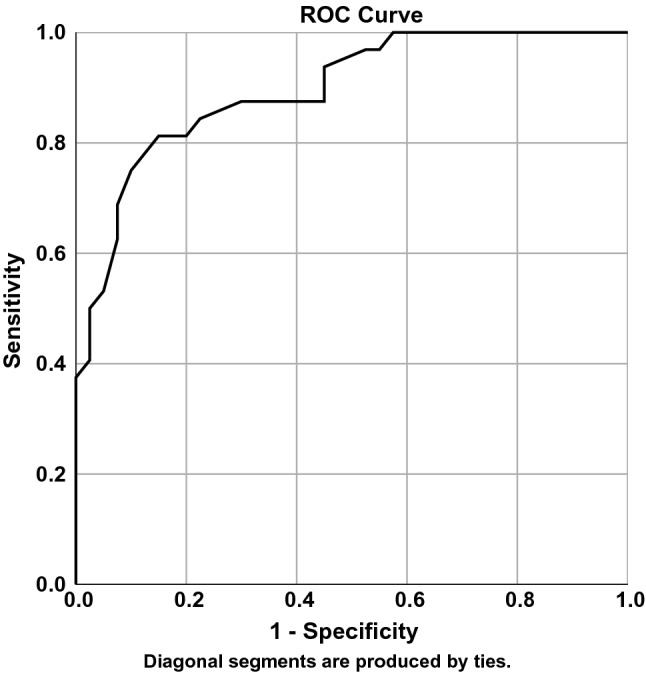
ROC-curve analysis of internal rotation with 90° hip flexion (IRF)

Due to their predictive ability in the linear regression, IRF, ERF, and ABD were further analysed with a ROC-curve analysis to explore their ability to detect an alpha angle > 60°. IRF had an AUC of 0.896 (Fig. 2), ERF 0.638, and ABD 0.679 (Table 4). ROM cut-offs with the highest sensitivity and specificity were identified. For IRF, the highest sensitivity and specificity was found at 27°, with sensitivity of 81% and specificity of 85%. For ERF and ABD, the cut-offs were 41° and 27°, respectively, with sensitivity of 72% and specificity at highest 60% (Table 4).

**Table 4:** Area under the curve (AUC) (95% CI), sensitivity, and specificity for range of motion (ROM) variables to detect alpha angles ≥ 60° (*n* = 72)

ROM	AUC (95% CI)	Degree	Sensitivity (%)	Specificity (%)
IRF	0.896 (0.825; 0.968)	27	81	85
ERF	0.638 (0.510; 0.766)	41	72	50
ABD	0.679 (0.552; 0.806)	27	72	60

*IRF* internal rotation with 90° hip flexion, *ERF* external rotation with 90° hip flexion, *ABD* abduction

## Discussion

The most important findings of the present study were that (1) less ROM in IRF was associated with a greater alpha angle in a sample of patients with longstanding hip and groin pain, and (2) that a 27° cut-off of IRF had good sensitivity and specificity to classify participants above or below the alpha angle threshold of 60°. Also, less IRF, ERF, and ABD were associated with a greater alpha angle, where 10° less ROM corresponded to an approximately 5° higher alpha angle.

A clinical assessment tool to help identify the presence of cam morphology could be of benefit in the diagnosis of FAIS, and ROM assessment is a low-cost and low-risk assessment that is routinely performed in this patient population. The association of ROM and the alpha angle found in the current study is in line with previous research with similar methodology. In one study, which used computed tomography to model and simulate ROM in people with FAIS, a 1° greater alpha angle corresponded to 0.46° less internal rotation in 90° hip flexion [1], which corresponds well to the results of the current study. In a cross-sectional study on a sample of 1021 Gaelic football players with FAIS, reduced ROM in all hip motions was associated with a greater alpha angle [3]. The authors only provided *p* values to describe the strength of the association, did not report correlation coefficients or beta-values, and the correlation analysis was not adjusted for sex [3]. In the current study, all ROM variables were also statistically significant prior to adjusting for sex. Moreover, a greater alpha angle appears to be associated with lower ROM in studies on asymptomatic athletic populations [18, 19, 22, 27]. Thus, the findings of the current study support the previously reported association between hip ROM in ABD, IRF and ER, and cam morphology, and can add to the clinical picture when examining patients with FAIS.

In the ROC-curve analysis, IRF displayed large AUC and good sensitivity and specificity to identify patients above and below the alpha angle threshold. A study by Kapron et al. performed a ROC-curve analysis between ROM and cam morphology on asymptomatic male American football players, defining a cam as > 50° alpha angle [19]. The authors reported a cut-off of 38° in internal rotation, measured with the patient seated in 90° of hip flexion, with a sensitivity of 81% and a specificity of 51% [19]. However, a cut-off of 50° could be considered a bit low for diagnosing cam morphology, as a cut-off of 60° appears to be best supported in the literature [23]. The different populations and alpha angle threshold may well explain the difference between the findings of Kapron et al. and those in the current study. While both ABD and ER were associated with ROM in the current study, IRF had better sensitivity and specificity. It appears that ROM assessment of IRF may provide valuable information about underlying hip morphology and can potentially guide decision making with regard to radiographic imaging.

There may be several reasons for limited ROM in an individual with longstanding hip and groin pain, such as sex, morphological variations, and perceived pain. Females generally have greater ROM than males [6], and males tend to have a higher alpha angle than women [29]. Morphological variations that may influence ROM include femoral torsion and acetabular version [4], which has been suggested to be present relatively frequently in people with hip-related pain [28]. Also, an association between thickness of the hip capsule and lower ROM has been reported [33]. In one study, patients with FAIS with more severe symptoms had significantly lower flexion ROM [9]. In another study, elite football players who had a previous time-loss injury to the hip or groin were found to have significantly lower ROM, regardless of cam morphology [41]. While the high alpha angle group in the current study had less ROM, HAGOS scores did not differ between groups, indicating that hip morphology is associated with ROM independently of perceived symptom severity.

The ROM assessment method used in the current study showed good to excellent intra-rater reliability and an SEM of 2.0°–4.3° for the different directions. These findings are similar to previous research [24, 38]. Both goniometers and inclinometers are commonly used in the literature to measure hip ROM. While both goniometers and inclinometers are reliable, significant differences in measurements between devices have been documented [38]. Therefore, the results of the current study may not be transferable to goniometer measurements. Another factor that influences the ROM of the hip is pelvic motion and position. Previous research has shown that significant pelvic movement in the sagittal plane occurs during passive flexion [20], and differences in the movement end-point and applied force might, therefore, influence measurement accuracy and reliability. Pelvic tilt has also been shown to influence rotation by changing relative acetabular version [36], leading to less internal rotation in an anterior pelvic tilt [36, 40]. In the present study, care was taken to stabilise the pelvis in a neutral position, but flexion may be more prone to measurement error as stabilisation depended on fixating the opposite limb rather than the pelvis itself. The findings of this study should be applied in clinical practice with consideration of the positioning of the patient and tools of assessment, as well as an awareness of the potential measurement error.

In clinical practice, ROM assessment is often conducted by visual estimation and perception of joint end feel. A recent study by Pålsson et al. reported moderate to high specificity for identifying patients with FAIS using a dichotomous (normal/decreased) evaluation of hip ROM in flexion, rotation, and abduction [34]. The findings of the current study indicate that using a more robust assessment method with a ROM cut-off may provide diagnostic information about the presence of underlying cam morphology in people with longstanding hip and groin pain.

In the current study, a frog-leg lateral projection and an alpha angle cut-off of 60° were used. Heterogeneity in the imaging projections and cut-offs in studies reporting on ROM in FAIS limit the possibility to compare the results of the present study to the results of previous research. The two published systematic reviews on physical impairments related to FAIS included studies of people with both cam and pincer FAIS, as well as people with isolated chondrolabral pathology [7, 10]. Also, many of the included studies did not adequately describe their radiographic investigation or cut-offs for the alpha angle in their methods. An anterior–posterior projection and a frog-leg lateral projection allow for the visualisation of cam morphology in different positions of the femoral head [5, 26]. Potentially, an osseous growth at different positions on the femoral neck may influence the hip joint and hip ROM differently [18]. Therefore, results on alpha angle and its potential association with ROM may be dependent on the radiographic projection and alpha angle cut-offs, and results from different methodologies may not be used interchangeably.

To our knowledge, this is the first study exploring the association of cam morphology and hip ROM in patients with longstanding hip and groin pain. It is also the first to examine whether a cut-off in ROM has merit as a test to identify an alpha angle above 60°. However, this study is not without limitations. The results should be interpreted with caution due to the exploratory nature of this study and the wide confidence intervals in the regression models. Although there was a significant association between ROM and alpha angles in this study, other factors that may contribute to ROM limitations, such as femoral torsion, acetabular version, pincer morphology, and capsular thickness, were not measured. Another limitation is the disproportionate number of men compared to women in the high alpha angle group. While steps were taken to minimise the effect of this imbalance by checking for multicollinearity and adjusting for sex in the linear regressions, the differences in sex may affect the results of our ROC-curve analysis by shifting the cut-off and over- or underestimating the diagnostic accuracy.

In the clinical setting, differential diagnosis of hip and groin pain can be a challenge. So far, ROM in patients with FAIS have primarily been compared to that of asymptomatic controls. The finding of the current study that greater cam morphology is associated with less ROM in a cohort of people with symptoms indicates the potential value of ROM testing to identify underlying morphology and informing differential diagnosis of hip and groin pain, especially in situations where imaging is not readily available.

Specifically, passive internal rotation of 27° or less may identify a patient likely to have cam morphology, and can help physical therapists or general practitioners deciding who to refer for further investigation and/or orthopaedic consultation.

## Conclusions

In the current study, less internal rotation, hip external rotation, and abduction were associated with a higher alpha angle in a population with longstanding hip and groin pain. IRF had the best sensitivity and specificity to identify cam morphology.

## Abbreviations

HAGOS: Copenhagen hip and groin outcome score
QoL: Quality of life
FLEX: Flexion
IRN: Internal rotation in neutral
IRF: Internal rotation in 90° flexion
ERF: External rotation in 90° flexion
ABD: Abduction
OA: Osteoarthritis
BMI: Body mass index
